# Co-Encapsulation of Drugs for Topical Application—A Review

**DOI:** 10.3390/molecules28031449

**Published:** 2023-02-02

**Authors:** Janaína Artem Ataide, Julia Cedran Coco, Érica Mendes dos Santos, Viviane Beraldo-Araujo, Jéssica Ribeiro Alves Silva, Karine Cappuccio de Castro, André Moreni Lopes, Nina Filipczak, Satya Siva Kishan Yalamarty, Vladimir P. Torchilin, Priscila Gava Mazzola

**Affiliations:** 1 Faculty of Pharmaceutical Sciences, University of Campinas (UNICAMP), Campinas 13083-871, SP, Brazil; 2 Center for Pharmaceutical Biotechnology and Nanomedicine, Northeastern University, Boston, MA 02115, USA; 3 Faculty of Chemical Engineering, University of Campinas (UNICAMP), Campinas 13083-852, SP, Brazil; 4 Department of Chemical Engineering, Northeastern University, Boston, MA 02115, USA

**Keywords:** nanoparticles, topical delivery, co-encapsulation, skin barrier

## Abstract

Achieving the best possible outcome for the therapy is the main goal of a medicine. Therefore, nanocarriers and co-delivery strategies were invented to meet this need, as they can benefit many diseases. This approach was applied specifically for cancer treatment, with some success. However, these strategies may benefit many other clinical issues. Skin is the largest and most exposed organ of the human body, with physiological and psychological properties. Due to its exposition and importance, it is not difficult to understand how many skin diseases may impact on patients’ lives, representing an important burden for society. Thus, this review aims to summarize the state of the art in research concerning nanocarriers and co-delivery strategies for topical agents’ applications targeting skin diseases. The challenge for the medicine of the future is to deliver the drug with spatial and temporal control. Therefore, the co-encapsulation of drugs and the appropriate form of administration for them are so important and remain as unmet needs.

## 1. Introduction

It is well known that skin is the largest and the most exposed organ of the human body, acting mainly as a barrier to the environment, and preventing the entrance of harmful microorganisms and substances and the loss of water [1,2]. Skin’s functions also include thermal regulation, sensations, and immune surveillance, besides being an important determinant of human identity and appearance [1]. Given its characteristics, it is easy to understand how conditions impairing skin’s integrity can affect patients’ general well-being [1,3]. Skin diseases affect almost one third of the world’s population and are the fourth most common cause of human diseases [1,4]. They are a heterogenous group of diseases that include acute and chronic conditions with infectious, congenital, degenerative, inflammatory, and cancerous causes [1,3]. For all of these conditions, treatment with topical agents may be beneficial, allowing high drug concentrations in the site, and potentially less toxicity when compared with systemic treatment [5].

Combination therapy involves the simultaneous use of two or more therapeutic agents, with different toxicities and acting through different mechanisms. Even though combination therapy gained prominence when better outcomes in cancer therapy were achieved [6,7,8], it is a common practice reported for different therapies [7,9], such as for hypertension [10], dyslipidemia [11], infectious diseases [12,13], neurological diseases [14,15], and even some skin diseases [5,16,17]. The main advantages of combination therapy are the potential synergistic or additive effects that show superior outcomes over the sum of individual drugs’ effects, allowing lower therapeutic doses that lead to a reduction in overall toxicity when compared with single-drug therapy [6,8]. 

Another way to enhance treatment options of various diseases is the use of nanostructures as drug delivery systems. Nanocarriers can improve drugs’ bioavailability, solubility, and permeability, increase drugs’ half-lives in the organism, and decrease the toxicity, as well as promote targeted or controlled release [18,19,20]. Even though different studies have already suggested the use of nanotechnology-based co-delivery systems, there are still challenges to be addressed, including some that are well known for single-drug encapsulation, such as loading efficiency and nanoparticles’ properties [18]. Other challenges are related to encapsulating drugs with different physicochemical properties and solubility, and regulating sequential release [8].

Studies have already reported the association of co-delivery and nanostructured systems for cancer treatment [8,21,22], and this strategy has also been used to develop topical treatments for skin diseases. Therefore, the aim of this review is to compare the nanostructured systems used for simultaneous encapsulation (co-delivery) of drugs, aiming at topical application.

## 2. Topical Drug Delivery: Challenges and Advantages

Skin is considered as a key site for local and systemic drug delivery. Its advantage is that it is easily accessible, which allows products from a wide range of formulations to be easily applied and then removed as needed. Skin is also relatively impermeable, which makes it a natural barrier. Thus, topical delivery systems have evolved from simple solutions and ointments to sophisticated nanotechnologies. The key factors for topical delivery products are to meet market requirements, including safety and sensory requirements, efficacy, and design [23]. 

Penetration of active substances through the skin is a very important issue in research on the effectiveness of compounds used in cosmetics and pharmacy. Obtaining the proper degree of penetration of active substances as well as confirming their clinical effectiveness are two of the key challenges for the modern cosmetics and pharmaceutical industry [24]. The effectiveness of any active substance is conditioned both by its impact on the lipids of the intercellular cement of the *stratum corneum* and on cell membranes. The main barrier that hinders the penetration of the active substance of the cosmetic through the skin is the *stratum corneum*, made of flat and keratinized cells [25]. It is the first and most important barrier to the penetration of active substances into the skin and to the target site. The structure of the *stratum corneum*, which is lipid in nature with a low water content, effectively regulates the degree to which the active substances penetrate the deeper layers of the skin. Considering the properties of the *stratum corneum*, lipophilic compounds with an admixture of hydrophilic properties are preferred [26]. 

**Figure 1:**
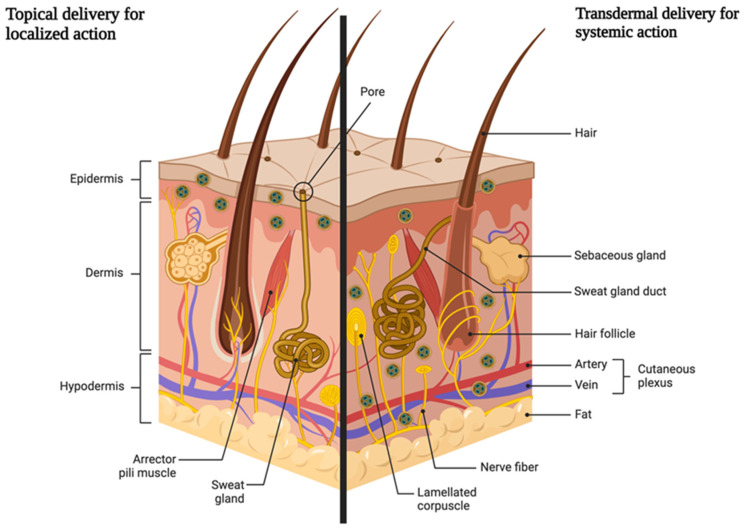
Schematic representation of two ways of drug delivery through the skin (created by Biorender).

As shown in Figure 1, skin is the barrier for two types of drug delivery: topical and transdermal. This paper puts specific focus on the topical delivery with localized action. The advantages of this type of the delivery include, but are not limited to:

- Application directly at the site of action. No first-pass effect and thus no reduction in the dose of the drug substance taken. The first-pass effect is defined as the metabolism of a part of an orally taken drug dose in the liver (or in the intestinal wall); because of which, the total amount of the active substance entering the bloodstream is lower.
- Reducing the risk of systemic effects and the possibility of side effects from the cardiovascular system, digestive system, or kidneys.
- Possibility of administration in people intolerant to oral doses for various reasons.
- Avoiding drug–drug interactions, for example with drugs that lower blood pressure or cardiological doses of acetylsalicylic acid.
- Possibility of reducing the oral dose [26,27].

**Figure 2:**
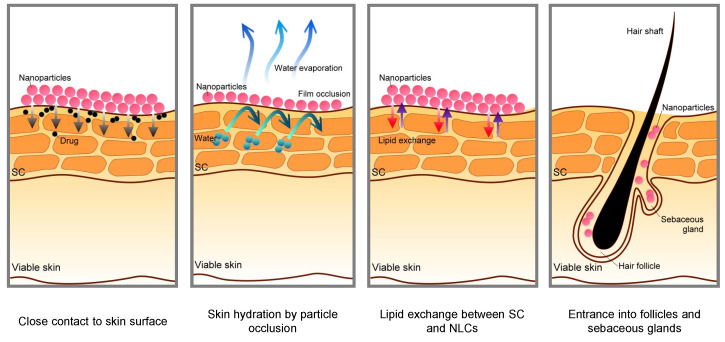
Schematic mechanisms for skin permeation of active components from nanoparticles [29].

To enable the effective transportation of biologically active substances into the skin, various types of innovative nanocarriers were created and used, in which small amounts of the active substance were enclosed. Depending on the structure and composition, the nanocarriers have a different penetration capacity and transfer the active substance to the target site, where it is then released [28]. Nanocarriers can enhance the permeation of the skin in few ways, as shown in Figure 2.

The biggest challenge for making nanocarriers for topical delivery is to make them biocompatible and nonirritant [30], and at the same time be able to encapsulate one or more active compounds. The encapsulation of the two drugs in one formulation would likely result in better patient compliance and treatment outcome. However, this can be challenging, especially with two drugs with extremely different polarities. This is why carriers with both a lipid and aqueous phases are of particular research interest [31].

## 3. Nanocarriers for Co-Encapsulation in Topical Delivery

### 3.1. Polymeric Nanoparticles

Polymeric nanoparticles are solid colloidal particles, which can be nanospheres, nanocapsules, dendrimers, polymeric micelles, and nanogels with a size ranging from 1 to 1000 nm [32,33,34]. The term nanoparticles is usually used to describe both nanocapsules and nanospheres. Nanocapsules have the therapeutic component localized in an aqueous or oily cavity surrounded by a polymer shell; in nanospheres, the drug is uniformly incorporated into the polymer matrix [34,35,36]. The organic phase’s composition and the method of preparation define the type of structure. The physicochemical characteristics and in vivo behavior of the generated nanostructure depend on the synthesis techniques used [36].

Conventional techniques for establishing polymeric nanoparticles include nanoprecipitation, solvent evaporation, supercritical fluid technology, dialysis, reverse salting out, and polymerization [34,35,37]. The polymers that are used can either be synthetic or natural, biodegradable or not [34]. Non-biodegradable polymers include poly(methyl methacrylate) (PMMA), polyacrylamide, polystyrene, and polyacrylates [38]. In particular, biodegradable polymeric particles such as poly(lactide), poly(lactide-co-glycolide) copolymers (PLGA), poly(ε-caprolactone), and poly(amino acids), as well as natural polymers such as chitosan, alginate, gelatin, and albumin, might change some kinetic patterns of drug release; in addition, they are more biocompatible and have lower toxicity [38,39]. 

The pharmacokinetic characteristics of polymeric nanoparticles, such as drug loading, controlled drug release, and structural stability in vitro and in vivo, are satisfactory [37]. Polymeric nanoparticles can deliver proteins, DNA, or RNA to a particular target organ or cell in addition to medicines. They are a good candidate for a carrier due to their extended circulation time in the body, biocompatibility, biodegradability, non-immunogenicity, and availability in a range of formulations and manufacturing techniques [32,34]. In addition to the benefits already mentioned, nanospheres and nanocapsules accumulate more in the *stratum corneum* than emulsion-type nanosystems. This allows the drugs to be released gradually and provides a satisfying period of retention in inflamed skin [32,40]. This type of nanocarrier has already been used for the co-delivery of topical agents for skin diseases (Table 1).

Yang et al. [42] created poly(lactide-co-glycolide) (PLGA) nanoparticles containing the antiretrovirals griffithsin (GRFT) and dapivirine (DPV) as long-acting topical products for pre-exposure prophylaxis for the prevention of HIV. The produced polymeric nanoparticles were almost entirely monodispersed, as evidenced by their PDI of 0.063, size of 184.3 nm, −23.4 zeta potential (mV), and encapsulation efficiency of 45.9% (griffithsin) and 69.4% (dapivirine). The presented results suggested that griffithsin and dapivirine encapsulation were independent, once encapsulation efficiency was similar for single-drug nanoparticles. Researchers discovered that the pH and the composition of the medium have an impact on how each antiviral drug releases from nanoparticles. Studies conducted on cells revealed that the antiretrovirals under investigation work synergically to prevent HIV-1 infection. The formed nanoparticles also allow for continuous release of the medications, which may encourage their administration on a weekly basis and increase adherence to treatment [42]. 

Co-loaded nanoparticles of hydroxytyrosol and hydrocortisone were prepared to minimize the adverse effects of hydrocortisone and provide additional anti-inflammatory and antioxidant benefits to atopic dermatitis treatment [41]. The authors studied the effects of varying the pH of chitosan solution between 3 and 7, and reported an influence in nanoparticles’ size, zeta potential, and encapsulation efficiency. The best formulation was achieved at pH 5, with a mean diameter of 235 nm, zeta potential of +40 mV, and encapsulation efficiency and loading capacity around 75% and 25% (hydrocortisone), and 55% and 30% (hydroxytyrosol). Optimized NP formulation was used for compounding an occlusive percutaneous formulation to continue studies. Co-delivery NP formulation significantly reduced hydrocortisone and hydroxytyrosol permeation across mice skin, and increased drugs’ retention in epidermis and dermis in an ex vivo permeation study, when compared with controls. Finally, in vivo effectiveness was assessed in NC/Nga mice with induced atopic dermatitis. Compared to the control group, co-delivery NP formulation significantly decreased transdermal water loss, erythema intensity, and dermatitis index. Consequently, the formulation demonstrated potential in the management of atopic dermatitis [41]. 

Shah et al. [40] developed a drug delivery system based on a nanogel system containing polymeric nanoparticles of spantide II and ketoprofen, both of which are anti-inflammatory. Chitosan and poly(lactide-co-glycolic acid) were used to make these nanocarriers, and oleic acid was used to modify their surfaces. The size and zeta potential of the surface-modified NPs changed when compared to the non-modified NPs, but encapsulation efficiency was not affected. The final NPs showed a diameter of 183 nm, zeta potential of 5.3 mV, and encapsulation efficiency of around 93% and 81% for spantide II and ketoprofen, respectively. The surface-modified NPs were then dispersed in hydroxypropyl methyl cellulose gel achieving the nanogel formulation. NP size in the nanogel was stable when stored under refrigeration, but it significantly increased when stored at 40 °C/75% RH (relative humidity). Co-delivery nanogels showed increased skin retention of spantide II and ketoprofen during in vitro permeation studies, and also improved in vivo treatment of allergic contact dermatitis and psoriasis mice models, suggesting the potential of this combination therapy to treat inflammatory skin disorders [40].

On the other hand, nanocapsules have also been produced aiming at co-delivery of topical agents. Lipid-core nanocapsules (LCNs) resemble vesicular carriers, formed by an oily inner core surrounded by a polymeric membrane [46,47], which make them suitable to encapsulate hydrophobic and hydrophilic drugs. Since the inner core is formed by lipids; LCNs have a high capacity of encapsulating lipophilic drugs, overcoming this drawback of liposomes [48].

Coradini et al. [43] developed LCNs for improved antioxidant effects of resveratrol and curcumin combination. For this, the authors physic-chemically characterized the co-encapsulation system and compared it with formulations containing each polyphenol. Separate encapsulation of each polyphenol increased their photostability and antioxidant activity; however, these parameters were even better when polyphenols were co-encapsulated. Co-encapsulation did not change nanotechnological characteristics and provided a controlled release of both agents. In a subsequent study, an increased delivery of resveratrol into deeper skin layers was observed in co-delivery LCN in comparison to individually encapsulated actives, making the formulation suitable for topical application in oxidative skin disorders [44].

A US patent US10850246B2 describes a new method for preparing ultra-small polymeric-lipid nanoparticles (USDNs). These nanoparticles are prepared via nanoprecipitation followed by layer-by-layer nanodeposition. The USDNs in the patent are pH sensitive with a pH-controlled release profile with their size ranging from 5–25 nm with loading capacity between 300–500 µg/mg. Efalizumab and ustekinumab were 22–72% released at pH 5.0 while no release was observed at pH 7.4 or above. Skin-penetrating properties were also evaluated, and after 2 h treatment, FITC-labeled USDNs were found in the epidermis diffused to dermal tissues. The authors believed that the small sizes of the USDNs made them effective in penetrating all of the skin strata and made them effective in delivering the payload topically [45].

### 3.2. Lipid Nanoparticles

Lipid nanoparticles play an important role as topical drug delivery systems mainly to treat skin disorders. Both solid lipid nanoparticles (SLNs) and nanostructured lipid carriers (NLCs) are colloidal lipid nanoparticles produced with lipids recognized as safe, tolerable, biodegradable, and having low skin irritability [49,50]. They also allow the incorporation of natural lipids or vegetable oils as excipients, giving extra functions to the formulation, e.g., antioxidant properties. NLCs are the second generation of lipid nanoparticles, which emerged to overcome the SLN limitation of drug expulsion during storage. In this case, there is a reorganization in the lipid matrix due to the crystallization of lipid molecules, which may provoke drug expelling. NLCs have at least one liquid lipid in their composition, having a low degree of matrix organization, which increases drug incorporation stability [51,52]. As for topical drug delivery, SLNs and NLCs are able to protect incorporated molecules from degradation, improving stability and skin penetration, and increasing skin hydration and occlusion, when incorporated into bases such as gels, lotions, and creams [50,53]. There are different methods of SLN/NLC production: high pressure homogenization is the most common, followed by sonication, solvent injection, emulsification, microemulsion, and others. They are versatile, being able to incorporate lipophilic and hydrophilic molecules [50]. The most significant results found in the literature on lipid nanoparticles for co-delivery are summarized in Table 2.

Jain et al. [57] encapsulated adapalene in NLC and incorporated it into a Carbopol gel with vitamin C for co-treatment of acne. Adapalene had a high entrapment efficiency in NLCs (87%), which showed good physicochemical parameters for skin administration (z-average 268.3 nm and polydispersity index 0.218) and stability for 3 months. The skin evaluation showed that the application of the drug with an antioxidant molecule improved the acne treatment regarding the non-encapsulated adapalene. The vehicle of both molecules improved the sustained drug release for up to 48 h.

Another strategy was proposed by Gupta et al. [61] to treat acne. They formulated isotretinoin and α-tocopherol acetate-loaded SLNs (size 193.4 nm, zeta potential −29 mV, EE 84% and 77%, respectively), incorporated in P407 gel and compared with the conventional gel in vivo. The co-incorporation of α-tocopherol acetate was a strategy to reduce the irritation induced by the acid portion of isotretinoin, which is very common when treating acne. Indeed, they found no irritability in rabbits’ skin after 72 h, compared to a slight and moderate erythema provoked by the conventional gel. An efficacy study was evaluated in a rat model of acne, where they detected no acne in epidermis and dermis, with a normal histological appearance, in contrast to the conventional gel (acne in dermis). 

Andrade et al. [59] efficiently encapsulated tacrolimus and clobetasol into NLC (EE > 90% for both drugs) and evaluated their influences on the lipid dynamic behavior of the NLC–lipidic matrix. They found, from EPR spectroscopy of spin labels, that nanoparticles were more rigid after incorporating the drugs. Furthermore, the oil from NLCs tended to remain on the superficial regions of the nanoparticle. According to the differences between the drugs (tacrolimus was more soluble in solid lipid, had a higher log P, and almost 2-fold the clobetasol molecular weight, while clobetasol was more soluble in liquid lipid), they were able to explain some aspects, such as their position in NLC, the faster release profile of clobetasol, and the positive influence of tacrolimus on clobetasol release in the first 24 h. On the other hand, clobetasol acted as a kind of permeation enhancer for tacrolimus, increasing more than 5-fold its skin permeation. After coating NLC with chitosan, Andrade et al. found that this biopolymer increased drugs’ permeation, reaching deeper skin layers, which favored the treatment in lupus discoid, in which there was a hyperkeratotic condition that meant difficult drug permeation and recovery from the disease.

Tacrolimus was also applied by Viegas et al. [60], giving a different approach. They developed NLCs for topical application, incorporating tacrolimus and TNFα-siRNA to treat psoriasis and inhibit the production of TNFα, a proinflammatory cytokine overexpressed in the psoriasis condition. They reached a stable formulation regardless of the polydisperse population (size lower than 300 nm and PDI > 0.3), with high EE (>90%) and a synergistic effect of both tacrolimus and siRNA, showing a decrease in TNFα synthesis at the basal level and improved treatment, reducing erythema in psoriasis plaques in mice.

Moreover, focusing on psoriasis treatment, betamethasone dipropionate and calcipotriol were incorporated into SLNs with the purpose of decreasing drugs’ limitations; betamethasone is common in psoriasis treatment as an effective antiproliferative agent, but it offers a risk of cutaneous atrophy and the rebound of psoriasis. Calcipotriol is a vitamin D derivative and has severe skin irritation potential. They found SLNs with size < 200 nm, low polydispersity (around 0.172), and high EE (around 85% for betamethasone dipropionate and 98% for calcipotriol). SLNs were non-irritant and presented a better distribution onto the skin layers when compared to the commercial product. From in vitro and in vivo assays of skin permeation and pharmacokinetic and skin irritation, the formulation also showed higher antipsoriatic efficacy than the commercial product [63]. 

Emphasizing the NLC ingredients approach, Ott et al. [58] co-encapsulated two different nature molecules: the hydrophilic antitumor pemetrexed and hydrophobic bio-flavonoid hesperidin. The purpose was to evaluate the behavior of different concentrations of squalene fractions, extracted from amaranth oil, in NLC production and molecule incorporation. Physicochemical characteristics, as described in Table 1, showed nanometric and monodispersed sizes, with high absolute zeta potential values and high entrapment efficiency of both molecules. Interestingly, in vitro antioxidant activity analysis showed that either free NLCs or molecule-loaded NLCs presented >90% activity on capturing free radicals. NLCs with hesperidin had a higher antioxidant activity (>95%), an expected result for a bio-flavonoid, indicated to prevent oxidative stress. The higher concentration of squalene fraction, the slower release of both molecules, reaching up to 40% and 80% after 24 h to pemetrexed and hesperidin, respectively. Curiously, they were able to entrap and to have a slow release of the hydrophilic pemetrexed, revealing the capacity of NLCs to incorporate both molecules in the small lipid vesicles in the nanoparticle core.

Venturini et al. [56] studied the co-encapsulation of imiquimod and copaiba oil in lipid nanostructures. In fact, copaiba oil has pharmacological application in the treatment of skin cancer due to its anti-inflammatory activity and the fact that it acts together with the immunomodulatory function of imiquimod in the treatment. However, copaiba oil was used as part of the formulation as well, to produce lipid capsules and NLCs based in Brazilian lipids. Therefore, they evaluated the characteristics of both nanostructures regarding imiquimod parameters (encapsulation efficiency, release profile, and pig skin permeation). This study was able to find that nanocapsules seemed to be more promising due to the higher drug retention in the different pig skin layers and due to controlled drug release.

Costa-Fernandez et al. [55] produced bioadhesive NLCs (NLC-chitosan or sodium alginate) to incorporate antioxidant agents (α-tocopherol and quercetin) and tea tree oil as antimicrobial agents to manage and treat wounds. They obtained nanoparticles between 307–330 nm with low polydispersity indexes (<0.25). The zeta potential varied according to the polymer used as the adhesiveness promoter. High encapsulation efficiencies were reached for both antioxidants (82–85% of quercetin and 92% of α-tocopherol). Incorporated tea tree oil did not diminish its antimicrobial effect but brought to the formulation vascular effects (bleeding) in Hen’s egg test (HET-CAM), which happened also with the non-encapsulated oil. NLCs stimulated cell migration (human fibroblasts), even without the encapsulated molecules, showing their intrinsic ability to improve the healing process and improve with the incorporated antioxidants.

Palliyage et al. [62] extensively studied SLNs as drug delivery systems to carry two poorly bioavailable molecules (curcumin and resveratrol) and to overcome their limitation to treat melanoma. With good physicochemical characteristics (particle size < 200 nm, PDI < 0.25, and zeta potential > −30 mV), SLN incorporated > 90% of curcumin and >60% of resveratrol. The molecules presented skin binding with shed snakeskin (≥70%) and sustained in vitro release, with a faster release of resveratrol (around 50% after 120 h) than curcumin (around 5% after 120 h), a difference that could be due to the higher solubility of curcumin in compritol 888, the lipid used to produce SLNs. As resveratrol is less soluble than curcumin in this lipid, it was released quicker. Nevertheless, the entrapped molecules were effective in avoiding SK-MEL-28 cell proliferation and reducing B16F10 melanoma cell migration. They also presented a synergistic action at all dose levels, revealing that a dose reduction for both molecules was possible.

Lacatusu et al. [54] produced NLCs with bioactive oils (raspberry seed oil or rice bran oil) to deliver indomethacin (a lipophilic non-steroidal anti-inflammatory) and willow bark extract (which contains salicin, a hydrophilic molecule with antioxidant and anti-inflammatory effects) to treat skin inflammatory diseases. They found nanoparticles with a low PDI (around 0.15 with a size of <140 nm), negative zeta potential (between −40 and −48 mV), and a surprisingly high entrapment efficiency for salicin (around 90%), which could be entrapped in the surfactants. Indomethacin had a lower EE (between 65 and 72%), but its release profile was 45% maximum, because of the high solubility in the lipids. An amplification of antioxidant activity was found in the formulation with both molecules, and it was non-toxic to L929 fibroblast cells (>90% viability), and flow cytometry analysis showed a low apoptosis induction (lower than 12%). The anti-inflammatory activation was dose-dependent. The choice to carry the nanocarrier in Carbopol hydrogel was to keep drug concentration for longer on the skin surface during the future treatment. 

Giving a different approach to lipid nanoparticles, Sakellari et al. [64] interestingly used SLN as a tool to stabilize w/o and o/w emulsions, via the Pickering mechanism, for the co-encapsulation of molecules with different hydrophilicity degrees. They showed success in the stabilization of both emulsions, which could be valuable for pharmaceutical, food, and other applications.

### 3.3. Liposomes

Liposomes are drug delivery systems that gained popularity over the past decades, reaching the market with liposomal anticancer and antifungal drugs [65]. Liposomes are vesicular structures, with one or more bilayers distributed around an aqueous core, that can encapsulate both hydrophilic and hydrophobic drugs [66]. This versatile feature is interesting for co-loading drugs of different polarities. For skin application, liposomes have been extensively researched due to their similarities, but may be disadvantageous due to their low viscosity [67]. The authors frequently resort to viscosity-modifiers to make the topical application feasible and enhance the residency in the skin. However, this often means a retardancy of release since there is another barrier to be surpassed. The most significant results found in the literature on liposomes and modified liposomes for co-delivery are summarized in Table 3.

Caddeo et al. [70] combined the natural polyphenols quercetin and resveratrol in liposomes for the treatment of skin cancer lesions. Liposomes were produced via direct sonication and were composed of soybean lecithin and oleic acid. The averaged vesicle size of liposomes was 79 nm with a mean PDI of 0.120, and ζ potential of −40 mV. Quercetin and resveratrol encapsulation efficiency (EE) were 71% and 72%, respectively, which remained stable for 2 months. Co-loading improved the accelerated stability, cellular uptake, and ROS scavenging ability when compared to single-drug liposomes. The dual-polyphenol liposomes also reduced the inflammation of skin lesions in mice. The authors suggested that the combination of polyphenols in liposomes is useful for oxidative and inflammatory skin disorders.

In another study, Eroğlu et al. [68] created a liposomal-based hydrogel with tetracycline and tretinoin as a combined treatment for acne. Liposomes were produced via the thin-film hydration method, followed by sonication. The formulation was optimized based on different phospholipids, the volume of round-bottom flasks, and sonication time. Co-loaded liposomes were composed of PC-DPPC and cholesterol, with a vesicle size of 166 nm, a mean PDI of 0.270, and ζ potential of +16 mV. The EE was 87% for tetracycline and 88% for tretinoin. No significant changes in size, PDI, ζ potential, or EE of co-loaded liposomes were observed during storage at 4 °C for 6 months. Aiming for a suitable topical application, co-loaded liposomes were incorporated into hydrogels and the pH, viscosity, and physical appearance were stable for 6 months. Regarding the drug release from liposomal-based hydrogels, 56% of tetracycline and 58% of tretinoin were released after 24 h. Tetracycline and tretinoin presented a synergistic effect against bacteria when co-loaded into the liposomal-based hydrogel, and inhibited biofilm formation.

Aiming for a novel combination therapy for psoriasis, Boakye et al. [71] developed erlotinib-loaded liposomes complexed with IL36α siRNA. Liposomes were composed of DSPC and a novel synthesized cationic amphiphilic lipid, tailored to increase the fluidity of the membrane and enhance the transdermal delivery. The averaged vesicle size was 132 nm with a mean PDI of 0.150 and ζ potential of +40 mV. Erlotinib encapsulation efficiency was 49% and complexation with IL36α siRNA was effective, due to the negatively charged siRNA electrostatic interaction with the cationic liposomes. Liposomes enhanced the permeation and deposition of drug and siRNA on the skin. In vivo, authors found the liposomal combination treated psoriatic-like plaques more efficiently than the single-drug or the commercially available formulation. The authors considered the combination to be a promising dual approach to treating psoriasis.

In a similar approach, Jose et al. [69] used cationic liposomes as a platform to co-deliver curcumin and STAT3 (signal transducer and activator of transcription 3) siRNA for skin cancer therapy. Cationic liposomes consisted of DOTAP, DOPE, C6 ceramide, and sodium cholate. The averaged vesicle size was 193 nm, with a PDI of 0.326 and zeta potential of +56 mV. The EE of curcumin was 87% and the siRNA successfully complexed with the positively charged liposomes. In vitro, the liposomal combination was superior to inhibit the growth of B16F10 cells when compared to curcumin or STAT3 siRNA alone, but no additive effect was observed. The co-loaded liposomes did not permeate deep into the skin via passive diffusion, but iontophoresis and intratumorally administration were able to successfully reduce the cancer growth in mice. The authors considered the iontophoresis of co-loaded liposomes a satisfactory and non-invasive approach to treating skin cancer.

Kohane et al. [72] developed a liposome formulation containing dexmedetomidine and tetrodotoxin for prolonging local ophthalmic anesthesia. The efficacy of a combination of drugs from different classes in prolonging eye anesthesia had already been established by the same authors. The liposomes were prepared using the thin-film hydration method with different concentration ratios of lipids. The authors determined that the best effect for prolonging eye anesthesia without toxicity was achieved using a 15:1 (DOPC:DOPG) ratio. This formulation presented liposomes with 446 nm of average size and PDI of 0.33. The encapsulation efficiency was around 64% and 44% for dexmedetomidine and tetrodotoxin, respectively. No data about drug release profiles or stability of formulation were shown by the authors. 

Ingebrigtsen et al. [82] created liposomes for acne treatment, containing benzoyl peroxide and chloramphenicol, via a novel manufacturing method—dual asymmetric centrifugation. Liposomes comprised of soybean lecithin, propylene glycol, and distilled water (2:1:2 *w*/*v*/*v* ratio) which were used for the entrapment of benzoyl peroxide (BPO) and chloramphenicol (CAM). Co-encapsulation of around 50% for BPO and 60% for CAM, respectively, was obtained and the size of the obtained liposomes ranged from 130 to 150 nm mean diameter, with a polydispersity index below 0.2. Liposomal formulation was less cytotoxic than the corresponding drug solutions used as references, hence demonstrating that DAC is a fast, easy, and suitable method for the encapsulation of more than one drug within the same liposomes.

#### Modified Liposomes

As seen, liposomes presented advantages as carriers to co-loading drugs. However, they are often referred to as having a rigid structure, meaning they are less likely to permeate the skin. Therefore, several studies are dedicated to studying modifications in liposomes’ structures that can ameliorate this drawback. In the co-loading for topical and transdermal delivery, the trend is to create ultra-deformable liposomes, also known as transferosomes or elastic liposomes. These modified vesicles can be created by modifying the composition with edge activators such as surfactants (e.g., sodium cholate, polysorbates, sodium dodecyl sulfate) that can interweave into phospholipid bilayers and make them more flexible [83]. This gained elasticity can make the vesicles surpass the *stratum corneum* more easily or penetrate the deeper layers of the skin, and so are considered more suitable for topical and transdermal delivery [77].

In fact, Lei et al. [75] demonstrated that transethosomes, a type of ultra-deformable liposome which combine alcohol and an edge activator in their composition, could enhance the permeation of co-loaded dacarbazine and tretinoin for cutaneous melanoma treatment, when compared to conventional liposomes. Multi-drug transethosomes were smaller (125 nm), with a narrower size distribution (PDI of 0.25) and higher ζ potential (−32 mV) than the liposomes (187 nm; PDI of 0.39; −18 mV). The EE was higher in transethosomes when compared to liposomes for both dacarbazine (67% versus 27%) and tretinoin (42% versus 21%). Transethosomes were two times more internalized than liposomes in B16-F10 cells and presented higher cytotoxicity towards cancer cells. In vivo, transethosomes enhanced the tretinoin and dacarbazine skin penetration by 2-fold and 3-fold, respectively, and enhanced the plasma concentration of dacarbazine by 3-fold when compared to liposomes. In this preliminary study, transethosomes were considered superior to conventional liposomes in all aspects studied as a dual approach for cutaneous melanoma.

Doppalapudi et al. [81] prepared ultra-deformable liposomes composed of DC-Chol, cholesterol, and sodium deoxy cholate to carry psoralen and resveratrol for vitiligo treatment. The size of liposomes was 126 nm with a mean PDI of 0.240, and ζ potential of +39 mV. Regarding drugs’ encapsulation, the EE showed values of 74% and 77% for psoralen and resveratrol, respectively. No significant change in PDI was observed when storing formulations at 4 °C for 30 days. Co-loaded liposomes showed a 65% and 73% release of psoralen and resveratrol in 18 h, respectively, which was very similar to one-drug-loaded liposomes. The authors found that co-loaded liposomes could act in vitiligo through a dual mechanism, stimulating melanin, tyrosinase activity, and antioxidant activity.

In another study, Cosco et al. [74] prepared ultra-deformable liposomes for the dual topical delivery of resveratrol and 5-fluorouracil, aiming at producing a novel skin cancer treatment. Ultra-deformable liposomes consisted of Phospholipon 90G and sodium cholate as the edge activator. Drugs were co-encapsulated in vesicles with an average size of 445 nm, with a mean PDI of 0.32, and ζ potential of −25 mV. The encapsulation efficiency of resveratrol and 5-fluoracil was 97% and 42%, respectively. Release from the multi-drug ultra-deformable liposomes after 24 h was 85% for resveratrol and 80% for 5-fluoracil. The authors observed an increase in the in vitro anticancer activity of the drugs in the multi-drug carrier when compared to the non-encapsulated or single-encapsulated drugs. The authors attributed this effect to the enhanced permeation of ultra-deformable liposomes in the skin, and the synergistic effect between the drugs, where 5-fluoracil-induced apoptosis is enhanced by the presence of resveratrol.

Dar et al. [78] developed ultra-deformable liposomes with amphotericin B and miltefosine for the topical treatment of cutaneous leishmaniasis. Liposomes were produced via the thin-film hydration technique followed by extrusion and were composed of Phospholipon 90G and polysorbate 80 as the edge activator. The mean size of the co-loaded liposomes was 140 nm, with an average PDI of 0.126 and ζ potential of −27 mV. The ultra-deformable liposomes presented 73% of elasticity, measured by the comparison before and after extrusion using a 50 nm polycarbonate filter. Multi-drug liposomes presented a high encapsulation efficiency of amphotericin B and miltefosine, showing values of 78% and 79%, respectively. In vitro antileishmanial activity was enhanced with 8-fold and 6-fold reductions of the IC_50_ of amastigotes when compared to amphotericin B and miltefosine solutions, respectively. The dual approach presented a synergistic effect in vivo, leading to full lesion healing and a reduced number of parasites in the infected mice.

### 3.4. Nanoemulsions

Microemulsions (MEs) and nanoemulsions (NEs) are lipid-based drug delivery systems composed of two immiscible phases, that are stabilized by surfactants and co-surfactants [84]. Micro/nanoemulsions are nomenclatures which are often interchanged in the literature. However, MEs and NEs differ in their stability and formation. MEs are thermodynamically stable and are formed spontaneously, with droplet sizes ranging from 100–400 nm, whereas NEs are thermodynamically unstable and require more energy to be formed, resulting in an emulsified system with droplet sizes from 10–100 nm [85,86]. The most significant results found in the literature on micro- and nanoemulsions for co-loading are summarized in Table 4.

Carvalho et al. [85] directly compared MEs and NEs for the co-loading of paclitaxel (PTX) and C6 ceramide (C6C) for the topical treatment of melanoma. NEs required an additional sonication step after vortex mixing, highlighting their difference from MEs, that were spontaneously formed. Dual-drug-loaded NEs and MEs presented similar average droplet sizes, PDI values, and ζ potential values (46 nm and 54 nm, PDI of 0.22 and 0.21, and −7 mV and −7 mV, respectively). NEs were considered safer than MEs in the irritation potential test and delivered more of the drugs into the skin with porcine skin models in diffusion cells. Thus, NEs were selected over MEs for further in vitro studies. The drugs presented a synergistic cytotoxic effect in melanoma cell lines, that was enhanced by their encapsulation into NEs, when compared to each drug solution or single-drug NEs. In 3D bioengineered models of melanoma, PTX-C6C-NEs disorganized the skin layers and promoted their permeation, but in a non-selective way, affecting healthy skin as well. The authors concluded that NEs’ formulation was superior to MEs’, but unspecific to the tumor lesions, and confirmed a synergistic effect that was facilitated by their co-loading into NEs.

MEs and NEs are prepared via emulsification with a mixture of water, oil, surfactant, and co-surfactants, often selected by solubility studies, to determine which components better solubilize the drug of interest. After this selection, a pseudo-ternary diagram is usually constructed to determine the component combinations that yield micro- and nanoemulsions [91]. However, one challenge in the co-loading of drugs in MEs and NEs may be finding a common ground of components that can solubilize all the drugs at the required amounts and yields of MEs or NEs.

In this scenario, Khatoon et al. [90] developed NEs for the co-delivery of three drugs, curcumin (CUR), resveratrol (RSV), and thymoquinone (TQN), aiming for psoriasis relief and a synergistic effect between the natural bioactive compounds. The authors screened 16 oils, surfactants, and co-surfactants to determine the best solubilizing agents for all of the drugs, and oleic acid, Tween 20, and PEG 400 were selected. Triple-loaded NEs presented a droplet size of 76 nm, with a mean PDI of 0.12. The cytotoxicity in psoriatic-like cells (A-431 cell line) of drug-loaded NEs was higher than the drugs in solution. In psoriasis-induced mice, NEs incorporated into a gel reduced the inflammation and skin lesions similarly to a commercial formulation for psoriasis and were more effective than the free drugs. NEs were incorporated into a gel to modify the viscosity of the formulation for a more suitable topical application. Although skin deposition was higher than that of drugs in solution, the ex vivo permeation of the drugs from the NEs gel was lower than NEs.

The authors often incorporate NEs and MEs into gels to make their rheological properties more favorable for topical application, such as better spreadability and adhesion [92]. The combination of MEs and NEs with gels may enhance the stability of the formulation and the permeation of drugs, reaching deeper layers of the skin [93]. However, this often means the drug permeation will be reduced, and this phenomenon is a drawback of the incorporation of NEs and MEs into aqueous bases.

For example, Zhang et al. [88] created an ME-based gel that enhanced the delivery and anti-nociceptive activity of two alkaloids, evodiamine (EVO) and rutaecarpine (RUT). These alkaloids are present in the fruits of *Evodia rutaecarpa*, the traditional Chinese medicinal plant Wu-Zhu-Yu. EVO- and RUT-loaded MEs presented an averaged droplet size of 71 nm, a mean PDI of 0.17, and ζ potential of −3 mV. The encapsulation efficiency of both bioactive compounds was near 100% (99% for EVO and 96% for RUT). MEs improved the transdermal drug permeation of both drugs by 2-fold, when compared to the drugs dispersed in a gel and the ME gel. The authors concluded that the gel network structures made the diffusion of drugs difficult and reduced the contact between the lipids from MEs and the lipids from the skin. ME gels with both drugs did not show irritation in vivo and reduced mice’s pain when compared to free drugs dispersed in the gel, by reducing inflammatory cytokine serum levels.

A study realized for Chattopadhyay et al. [94] developed a nanoemulsion containing diacerein (DC) and glucosamine sulfate (GS) that was mixed with a hydrogel prepared with xanthan gum (XG) forming a nanoemulsion-laden hydrogel (NLH) for osteoarthritis treatment. The NEs average hydrodynamic diameter was 81.95 nm, and its PDI value was 0.285. The ζ potential was −39.33 mV and this negative zeta potential value prevented particle aggregation as a result of electric attraction between the charged globules, which improved storage stability. Additionally, the negative zeta potential value may contribute to better drug permeation through the skin [95]. For DC and GS in the NLH, the percentage drug content values were 99.36 and 98.99, respectively. The NLH had an average pH of 6.05, which is similar to skin’s pH. Osteoarthritis was induced in Wister rats using the monosodium iodoacetate (MIA)-induced model and, for 3 weeks, 200 mg of NLH was applied twice to the right knee in these animals The total levels of GS and DC that diffused throughout the skin for the XG hydrogel system were 75% and 82%, respectively. Rabbits were used for the skin irritation study and the examined animals experienced nearly no skin reactions in the initial skin irritation trial, including neither erythema nor edema or any other kind of rash. The NLH formulation remained physically stable during the period, and no significant changes in droplet size, drug permeability, or the percentage of uniform drug content were seen. The authors found that the co-delivery of DC and GS in an NE loaded with transdermal hydrogel for osteoarthritis caused chondroprotection with a decrease in inflammatory biomarkers in a monosodium iodoacetate-induced experimental osteoarthritis model.

Overall, ME and NE may be an effective way to enhance the delivery of drugs through the skin and enhance the stability of the formulation. Still, they can be challenging to formulate and may need additional incorporation into gels for topical delivery, which can lead to drug delivery retardancy.

### 3.5. Niosomes

Niosomes (NIOs) are vesicular nanocarriers with lamellar structures composed of amphiphilic molecules surrounded by an aqueous compartment. These amphiphilic molecules, known as surfactants, contain hydrophilic groups (heads) and hydrophobic groups (tails), since in an aqueous environment they exhibit self-assembly properties, being able to co-encapsulate hydrophilic and hydrophobic drugs [96,97]. These nanocarriers are interesting as DDS, once they can present biodegradable, biocompatible, and non-immunogenic characteristics. Moreover, NIOs have a long shelf life, exhibit high stability, and can allow drug delivery to the target site in a controlled and/or sustained manner. In addition, NIOs improve the solubility and oral bioavailability of poorly soluble drugs, as well as the skin penetration of drugs that are applied topically [97,98]. The most significant results found in the literature on the co-delivery of drugs applied to topical delivery employing niosomes are summarized in Table 5.

Yang et al. [100] developed NIOs composed of ceramide to carry methotrexate (MTX) and nicotinamide (NIC) for psoriasis treatment. The averaged particle size of NIOs was 181 nm with a mean PDI of 0.050, and ζ potential of −24.5 mV. Regarding drug encapsulation, the EE was 71% for MTX. The authors demonstrated that the nanoformulation (MTX/NIC-NIOs) presented the strongest anti-proliferation effect, decreasing the cell viability to 62.62%, which was significantly lower than the other samples (i.e., 84.11% for blank NIOs, 76.09% for free MTX, and 71.80% for MTX/NIC solution). Moreover, MTX/NIC-NIOs increased the effectiveness of MTX treatment, once it was observed that compared with MTX oral administration, the topical administration of MTX/NIC-NIOs acted on the skin lesions. In addition, both NIC and ceramide from MTX/NIC-NIOs contributed to ameliorating skin lesions. Therefore, compared to MTX oral administration, the MTX/NIC-NIOs group exhibited superior performances in ameliorating skin lesions. Overall, the psoriasis area severity index (PASI) scores decreased remarkably in the order of MTX/NIC-NIOs < NIC solution < MTX (oral) < MTX suspension < MTX/NIC solution < blank NIOs. In vitro and in vivo permeation studies showed that NIOs significantly increased the permeation of MTX and NIC through and into the skin and increased the ability of these drugs to reduce the mRNA level of imiquimod (IMQ)-induced pro-inflammatory cytokines, making it evident that the combination of drugs had a synergistic effect on the treatment of psoriasis.

In another interesting study, Tavano et al. [99] prepared NIOs composed of polysorbate 60 as a commercial surfactant to carry resveratrol (RES), α-tocopherol (α-TOC), and curcumin (CUR) for the transdermal delivery of antioxidant molecules for the prevention of diseases caused by oxidative stress. The averaged particle size of NIOs ranged between 471 and 565 nm, with a mean PDI of less than 0.250. The co-encapsulation efficiency of α-TOC and CUR was 5.56 × 10^−7^ and 5.83 × 10^−7^ mol, respectively, while the co-encapsulation of RES and CUR was 4.08 × 10^−7^ and 4.18 × 10^−7^ mol, respectively. The permeation through the skin in the period of 12 h for samples containing CUR and RES was 93 and 19%, respectively. In the samples containing α-TOC and CUR, the permeation was 94 and 12%, respectively. The results showed synergistic activity between the co-encapsulated antioxidants. The nanoformulation containing RES/CUR showed a percentage of inhibition of free radicals of 40%, while in the formulation containing α-TOC/CUR, the inhibition reached 100%. However, the RES/CUR samples ensured an optimal performance in terms of the cumulative number of antioxidants permeated across the skin. The authors concluded that both nanoformulations showed a promising transdermal delivery of antioxidant molecules.

### 3.6. Polymeric Micelles

Polymer micelles (PMs) are DDS, usually between 10 and 100 nm, composed of amphiphilic block copolymers with hydrophilic and hydrophobic blocks self-assembled in aqueous solution, usually with a spherical structure, and characterized by a core hydrophobic inner shell and a hydrophilic outer shell [101]. PMs have a high load capacity, solubilization power, and stability in the bloodstream, which make them potentially promising in studies carried out with the possibility of greater efficacy and a superior safety profile [101,102]. The most significant results found in the literature on the co-delivery of drugs applied to topical delivery employing polymeric micelles are summarized in Table 6.

In this context, Sharma et al. [103] developed a phospholipid-based mixed PM for the co-delivery of isotretinoin (ITR) in combination with clindamycin phosphate (CLIN) for the treatment of acne vulgaris. The averaged particle size of micelles was 19.30 nm, with a mean PDI of 0.110, and ζ potential of −3.12 mV. The drug content of nanoformulation displayed a good encapsulation parameter: 99.9 and 100% for ITR and CLIN, respectively. The order of CLIN deposition in the skin was as follows: ITR/CLIN-loaded PM gel (14.4%) > free CLIN dispersion (11.9%) > marketed clindamycin phosphate formulation Clindac-A^TM^ (9.1%). The same behavior was also observed for ITR deposition in the skin, as follows: ITR/CLIN-loaded PM gel (13.5%) > free ITR drug dispersion (10.4%) > marketed isotretinoin formulation Sotretgel^TM^ (6.2%). The nanoformulation not only decreased the minimum inhibitory concentration (MIC) of the ITR but also helped to retain the drug in the desired location in the skin layers, in addition to demonstrating the antimicrobial activity of the ITR against Propionibacterium acnes.

In another interesting study, Xu et al. [104] prepared mPEG-PLA PMs to load both timolol (TM) and latanoprost (LTP), followed by mixing in 2-hydroxyethyl methacrylate monomer to obtain contact lenses for the treatment of glaucoma. The averaged particle size of micelles was 20.96 nm with a mean PDI of 0.068. The EE showed values of 93.2 and 74.8% for TM and LTP, respectively. An in vivo pharmacokinetic study of PM-loaded contact lenses showed a TM concentration of 33.08 μg/mL at 0.25 h, remaining detectable in tears for up to 120 h, and the LTP showed a sustained drug release in tears for 96 h, with a maximum drug concentration of 0.39 μg/mL at 0.083 h, suggesting an improved drug residence time and greater bioavailability capacity of both drugs. The study displayed the successful application of PM-loaded contact lenses for sustained release of the drugs with a minimal impact on contact lens properties, showing no obvious histopathological changes in the conjunctiva and cornea when used for 144 h. Contact lenses were shown to be safe and compliant with ocular drug release requirements.

Patel et al. [105] prepared hydrogel composed of poly(L-lysine-b-L-phenyl alanine) (PLL-PPA) and poly(L-glutamic acid-b-L-phenylalanine) (PGA-PPA) to carry curcumin and amphotericin B for wound healing. The averaged particle size of PLL_100_–PPA_5_ was 196 nm, and for PLL_100_–PPA_5_ it was 173 nm, with a mean of PDI of 1.23 for PLL_100_–PPA_5_ and 1.15 for PGA_100_–PPA_5_. The ζ potential was −68 for PLL_100_–PPA_5_ and −63 for PGA_100_–PPA_5_. Concerning drug encapsulation, the EE showed values of 76.5% and 87.4% for PLL–PPA and PGA–PPA, respectively. The drug release from the micelle-hydrogel was evaluated in various pH conditions (pH 3, 7, and 11) and demonstrated that the drug-loaded composite was sensitive to pH changes. At pH 11, the cationic PLL-PPA micelles displayed a hastened release of Cur; at lower pH levels, the release was more gradual. PGA-PPA micelles, however, demonstrated the reverse pattern, exhibiting a burst release at pH 3 and a protracted release profile at a higher pH. Micelle hydrogel composites with a high surface charge (both positive and negative) exhibited rapid Amp B release, while a low net charge displayed sustainable release. This burst release of antibiotic and/or antifungal drugs in the first phase of wound healing may be advantageous for preventing sepsis in the exposed wound, whereas sustained release may facilitate wound closure. These findings suggest that the micelle–hydrogel composite could be used as a dual drug release technology due to its outstanding adjustable characteristics and regulated multidrug release.

## 4. Microneedles

The *stratum corneum* is the first and main obstacle for the topical delivery route, which can be bypassed by microneedles (MNs). MNs were first described in a United States patent and defined as a drug delivery device with a drug reservoir and multiple projections, that can penetrate the *stratum corneum*, used in percutaneous administration of drugs for local or systemic therapy [106]. Since then, patches, rollers, and devices have been developed applying MNs, proving claims to enhance transdermal delivery, be minimally invasive, cause no pain upon administration, and be capable of being self-administered [107,108,109]. Microneedles can be made of metal, silicon, glass, ceramic, carbohydrates, or polymers, and are typically classified into four types: solid, coated, dissolving, and hollow and hydrogel, which have already been reviewed elsewhere [108,109,110]. The most significant results found in the literature on the co-delivery of drugs applied to topical delivery employing microneedles are summarized in Table 7.

A core-shell MN was developed by Yang et al. [111] to effectively co-encapsulate biomacromolecules and small drug molecules. In this study, an anti-PD-L1 antibody and 1-methyl-D,L-tryptophan, which already have synergistic anti-tumor effects in melanoma, were used. The shell of MN was composed of chitosan electrostatically bound to the antibody, and the core of polyvinyl alcohol (PVA) hydrogen-bonded with methyl-tryptophan. After development, the antitumor effect of drug-loaded core-shell MN was evaluated in subcutaneous melanoma mice models, presenting a slower tumor growth rate than those treated with an intra-tumor injection of combination drug solution.

Triamcinolone acetonide and verapamil were co-loaded in dissolving microneedles to treat hypertrophic scars. Due to its hydrophobic nature, triamcinolone was previously complexed with hydroxypropyl β-cyclodextrin to improve its solubility in the mixture of carboxymethyl chitosan and *Bletilla striata* polysaccharide used in MN fabrication. MNs were completely dissolved in 40 min, delivering a model drug at the same time, meeting the rapid dissolving requirements for hypertrophic scar application. Its efficacy was also tested using the rabbit hypertrophic scar model, showing a decrease in scar thickness and the expression of hydroxyproline and TGF-β1 [112].

More recently, MNs have been used to mediate the delivery of drug-loaded nanoparticles, helping them to transpose the upper skin layer [117]. For example, MNs were loaded with two different nanoparticle formulations to promote hair regrowth [113]. For this, first, rapamycin was encapsulated in PLGA nanoparticles, while epigallocatechin gallate was crosslinked with keratin to form nanoparticles. Nanoparticles were spherical in SEM images, with nanoparticles with sizes of 150 and 100 nm, and loading capacities of 82% and 76% for rapamycin and epigallocatechin, respectively. Nanoparticles were then mixed and used for hair regrowth after incorporation into a dissolving PVP (polyvinylpyrrolidone) MN. PVP microneedles showed good mechanical properties and were able to penetrate the skin, dissolving within 60 s. MNs were proven to be biocompatible once macrophages and T cell markers were not significantly expressed in mice after application. In vivo studies in C57BL/6J mice also showed that nanoparticle-loaded MNs significantly improved hair growth. Another example of nanoparticles’ incorporation in microneedles was developed by Peng et al. [114] for the local treatment of melanoma. Single-phase or biphasic porous PLGA nanoparticles can be formed by the addition in the organic phase or in both (organic and aqueous) phases, respectively. These porous PLGA nanoparticles were used to incorporate paclitaxel and indocyanine green for chemo-photothermal combined therapy. Developed nanoparticles presented a mean diameter between 142–152 nm, a PDI of 0.13 and 0.18, and positive zeta potential between 12–13 mV. In single and biphasic NPs, paclitaxel encapsulation efficiency was above 90%, while indocyanine green was around 75 to 76%, respectively. Despite not showing a difference in encapsulation efficiency of paclitaxel, biphasic and single-phase porous nanoparticles showed a difference in their release, reaching 82% and 69% in 72 h, respectively. Biphasic porous NPs showed higher cytotoxicity than single-phase NPs, which was attributed to a more intense tubulin polymerization. To prepare the MNs, NPs were mixed with hyaluronic acid and a four-step casting process was used. After that, the mold was filled with a mixture of hyaluronic acid and hydroxypropyl-β-cyclodextrin. To fabricate the support film, polyvinylpyrrolidone K90 was used. The drug loading assay demonstrated that all MN formulations (with different NPs incorporated) contained an equal amount of paclitaxel and indocyanine green. MNs were tested in vivo using a melanoma mice model, showing superior results for MNs carrying biphasic porous nanoparticles and a synergistical effect of chemotherapy and photothermal therapy. Animal imaging also proved drug retention in the tumor site, avoiding entering systemic circulation [114].

An international patent WO2022119985A1 describes dissolvable microneedle arrays loaded with curcumin–albumin extracellular vesicles for the control of skin inflammation. Curcumin stability increased after co-incorporation with albumin in the extracellular vesicle, keeping 90% and 45% of curcumin initial content when kept in PBS at 37 °C for 3 h and 7 days, respectively. MNs dissolved within 5 min after application into rat skin, and thus can be considered as a burst release system. After incorporation into MNs, the cellular uptake and anti-inflammatory activity of curcumin–albumin extracellular vesicles did not change, and bioactivity was maintained after 12 months when stored at room temperature [115,116].

## 5. Skin Diseases That Could Benefit from Co-Delivery

As already mentioned, skin diseases are a heterogenous group and can be caused by many conditions, such as genetic conditions, microorganisms’ infections, wounds and burns, inflammation, and cancer [1,3,4]. Among skin diseases, atopic dermatitis possesses the highest disease burden, ranking 15th among all non-fatal diseases [4,118]. Atopic dermatitis is a chronic, relapsing, inflammatory and pruritic condition, which is more prevalent in early childhood, followed by middle-aged and older populations [118]. Its pathology is complex, and is still under investigation [89], including compromised skin barrier integrity, immune dysregulation, and genetic, environmental, and lifestyle factors [118,119]. Atopic dermatitis is mostly treated with a combination of topical agents from several classes, even in severe cases that need association with systemic agents or phototherapy [16,120]. 

Psoriasis is an immuno-mediated inflammatory skin disease, which is prevalent and incident worldwide, and related with a high patient burden [4,121,122]. The pathology of psoriasis is also complex and multifactorial [123]; however, its most-known signal is sustained inflammation, with uncontrolled keratinocyte proliferation and differentiation [17]. However, the inflammatory state may not be restricted to the skin, leading to other comorbidities such as arthropathy, and psychological, cardiovascular, and hepatic diseases, which lead some authors to classify psoriasis as a systemic disease [17,121]. Its treatment depends on disease severity and associated comorbidities, and can include topical therapy with a combination of glucocorticoids, vitamin D, and phototherapy in mild to moderate cases, or systemic treatments in severe cases [17].

Cancer burden and prevalence worldwide is well known and established. In 2020, 19.3 million new cancer cases occurred worldwide, leading to almost 10 million deaths [124]. Skin cancers are the most frequent solid cancers [125], with increased incidence but stable mortality rates [124,126]. An increased risk of skin cancer development has been associated with high UV light exposition [2,127]. Even though surgery for tumor removal has been the preferred treatment for skin cancers and is extremely effective in most cases, topical treatments may appear as an alternative for some cases [5,128].

## 6. Conclusions and Future Perspective

The increased popularity of topical medications in recent years is impressive. This is mainly because this form has proven to have more advantages than disadvantages. After all, the skin is ideal for drug delivery because it can produce both systemic and local effects. Local drug delivery systems have certainly changed the way we look at drugs. More and more medical institutions and healthcare professionals are adopting this form of treatment to improve their services to patients. This breakthrough in medicine provides a future of healthcare that is more effective and enjoyable for patients. To achieve this goal, the co-encapsulation of two or more active substances is one of the ways. Co-encapsulation is not only the art of combining molecules, but also the craft of achieving synergism through reciprocity. The term reciprocity in the case of co-encapsulated molecules means cohabitation within nanocarriers that is not compromised by molecular interactions, at least those that negatively affect their therapeutic effect or give rise to any cross-resistance, while maintaining adequate molecular integrity through synchronized release with the possibility of synergy. In other words, the goal of co-encapsulation is not just to achieve co-delivery, but rather to deliver it to the right place, at the right dose, and at the right time. This is where the greatest challenge for future medicine lies. Thus, there is a need to characterize the co-encapsulated molecules very precisely and find the right dosage form for them.

## Figures and Tables

**Table 1:** Examples of polymeric nanoparticles and the diseases that can benefit from co-encapsulated molecules.

Composition	Preparation Method	Active Encapsulation	Indication	Reference
Chitosan	Ionic crosslinking	Hydroxytyrosol and hydrocortisone	Atopic dermatitis	[41]
PLGA	Emulsion solvent evaporation	Griffithsin and dapivirine	HIV	[42]
Chitosan and PLGA	Emulsion solvent evaporation	Spantide II and ketoprofen	Inflammatory	[40]
PCL, sorbitan monostearate, grape seed oil, polysorbate 80	Precipitation of preformed polymer	Resveratrol and curcumin	Oxidative stress	[43,44]
DSPC, PLGA, chitosan	Nanoprecipitation followed by layer-by-layer nanodeposition	Efalizumab and ustekinumab	Psoriasis	[45]

PLGA: poly-(lactide-co-glycolic acid); HIV: human immunodeficiency virus; PCL: poly(ε-caprolactone), DSPC: 1,2-distearoyl-sn-glycero-3-phosphocholine.

**Table 2:** Examples of the lipid nanoparticles and the diseases that can benefit from co-encapsulated molecules.

Composition	Preparation Method	Active Encapsulation	Indication	Reference
Glyceryl monostearate, cetyl palmitate, raspberry seed oil or rice bran oil, phosphatidylcholine, polysorbate 20, poloxamer 188	High-pressure homogenization	Indomethacin and willow bark extract	Inflammatory	[54]
Shea butter, argan oil, sorbitan monooleate, polysorbate 80, poloxamer 407	Hot homogenization–sonication	α-tocopherol, quercetin, and tea tree oil	Wound	[55]
NCC: PCL, sorbitan monostearate, polysorbate 80 NBL: cupuaçu seed butter, sorbitan monostearate, polysorbate 80	NCC: interfacial deposition NBL: high-pressure homogenization	Imiquimod and copaiba oil	Cancer	[56]
Tristearin, labrasol, phospholipid-90NG, polysorbate 80, incorporated in Carbopol 934 gel	High-pressure homogenization method	Adapalene and vitamin C	Acne	[57]
Glycerol monostearate, cetyl palmitate, amaranth oil, sodium collate, polysorbate 20, synperonic PE, poloxamer F68	High-shear homogenization coupled with high-pressure homogenization	Pemetrexed and hesperidin	Cancer	[58]
Lecithin, taurodeoxycholate, stearic acid, oleic acid	Microemulsion	Tacrolimus and clobetasol	Lupus	[59]
Glycerol distearate, oleic acid, polyethylenimine, poloxamer P407	Hot homogenization and sonication	Tacrolimus and TNF-α SiRNA	Psoriasis	[60]
Glyceryl monostearate, polysorbate 80, incorporated in poloxamer P407 gel	Microemulsion	Isotretinoin and α-tocopherol	Acne	[61]
Compritol 888, poloxamer 188, polysorbate 80	High-shear homogenization	Curcumin and resveratrol	Cancer	[62]
Precirol ATO 5, poloxamer 188	Hot-melt high-shear homogenization	Betamethasone dipropionate and caleipotriol	Psoriasis	[63]

NCC: nanostructured copaiba capsule; PCL: poly(ε-caprolactone); NBL: nanostructured Brazilian lipids; TNF-α: tumor necrosis factor; siRNA: small interfering RNA.

**Table 3:** Examples of the liposomes and modified liposomes and the diseases that can benefit from co-encapsulated molecules.

Nanocarriers	Composition	Preparation Method	Active Encapsulation	Indication	Reference
Liposomes	PC-DPPC and cholesterol	Thin-film hydration technique followed by sonication	Tetracycline and trans-retinoic acid	Acne	[68]
	DOTAP, DOPE, C6 ceramide, and sodium cholate	Thin-film hydration technique followed by extrusion	Curcumin and siRNA	Cancer	[69]
	Lipoid S75 and oleic acid	Hydration followed by sonication	Resveratrol and quercetin	Inflammatory	[70]
	Cationic pyrrolidium lipid, DSPC, CTAB, and cholesterol	Thin-film hydration technique followed by sonication	Erlotinib and IL36α siRNA	Psoriasis	[71]
	DOPC, DOPG, and cholesterol	Thin-film hydration	Dexmedetomidine and tetrodotoxin	Ophthalmic anesthetics	[72]
Modified Liposomes	Phospholipon 90G, cholesterol, and cyanur-PE	Thin-film hydration technique followed by sonication and extrusion	Vancomycin and lysostaphin	Antimicrobial	[73]
	Phospholipon 90G, cholesterol, and sodium cholate	Thin-film hydration technique followed by sonication	Resveratrol and 5-fluorouracil	Cancer	[74]
	Phospholipid and sodium dodecyl sulphate	Thin-film hydration technique followed by sonication	Dacarbazine and trans-retinoic acid	Cancer	[75]
	Phospholipon 90G and sodium cholate	Thin-film hydration technique followed by extrusion	Bergamot essential oil and ammonium glycyrrhizinate	Inflammatory	[76]
	Phosphatidylcholine, cholesterol, and cetylpyridinium chloride	Thin-film hydration technique followed by sonication	Meloxicam and quercetin	Inflammatory	[77]
	Phospholipon 90G and polysorbate 80	Thin-film hydration technique followed by extrusion	Amphotericin B and miltefosine	Leishmaniasis	[78]
	Phospholipon 90G and polysorbate 80	Thin-film hydration technique followed by extrusion	Sodium stibogluconate and ketoconazole	Leishmaniasis	[79]
	Phospholipon 90G and polysorbate 80	Dropwise injection of organic phase into aqueous phase	Rifampicin and vancomycin	Leishmaniasis	[80]
	DC-chol, cholesterol, and sodium deoxy cholate	Thin-film hydration technique	Psoralen and resveratrol	Vitiligo	[81]

PC-DPPC: 1,2-Dipalmitoyl-sn-glycero-3-phosphocholine; DOTAP: 1, 2-Dioleoyl-3-trimethylammonium propane; DOPE: 1, 2-dioleoyl-sn-glycero-3-phospho-ethanolamine; siRNA: small interfering RNA; DSPC: 1,2-Distearoyl-sn-glycero-3-phosphocholine; DOPC: 1,2-dioleoyl-sn-glycero-3-phosphocholine; DOPG: 1,2-dioleoyl-sn-glycero-3-phospho-rac-(1-glycerol); DC-chol: 3ß-[N-(N′, N′-dimethylaminoethane)-carbamoyl] cholesterol hydrochloride.

**Table 4:** Examples of micro- and nanoemulsions and the diseases that can benefit from co-encapsulated molecules.

Composition	Preparation Method	Active Encapsulation	Indication	Reference
Squalene, Pluronic F68, and Myverol 18-04K^®^	Addition of aqueous phase into organic phase, followed by mechanical homogenization and sonication	Tretinoin and tetracycline	Acne	[87]
Ethyl oleate, cremophor EL, PEG 400, hyaluronic acid, and water	Dropwise addition of aqueous phase into organic phase	Evodiamine and rutaecarpine	Analgesic	[88]
Isopropyl myristate, phospholipid, ethanol, and polysorbate 80	Dropwise addition of aqueous phase into oil phase under magnetic stirring	Fluticasone propionate and levocetirizine dihydrochloride	Atopic Dermatitis	[89]
Polysorbate 80, tributyrin, oleic acid, tricaprylin, water, and Poloxamer 407	Vortex mixing and sonication	Paclitaxel and C6 ceramide	Cancer	[85]
Oleic acid, polysorbate 20, PEG 200, and water	Aqueous titration method	Curcumin, resveratrol, and thymoquinone	Psoriasis	[90]

PEG: polyethylene glycol.

**Table 5:** Examples of niosomes and the diseases that can benefit from co-encapsulated molecules.

Composition	Preparation Method	Active Encapsulation	Indication	Reference
Polysorbate 60	Film hydration method	Resveratrol, α-tocopherol, and curcumin	Oxidative stress	[99]
Ceramide	Ethanol injection method	Methotrexate and nicotinamide	Psoriasis	[100]

**Table 6:** Examples of polymeric micelles and the diseases that can benefit from co-encapsulated molecules.

Composition	Preparation Method	Active Encapsulation	Indication	Reference
Phospholipids	Self-assembly method	Isotretinoin and clindamycin	Acne	[103]
mPEG-PLA	Thin-film hydration method	Timolol and latanoprost	Glaucoma	[104]

mPEG-PLA: methoxy poly(ethylene glycol)-poly(lactide) copolymer.

**Table 7:** Examples of microneedles formulated with combined drugs or for the delivery of nanoparticles and the diseases that can benefit from co-encapsulated molecules.

Composition	Preparation Method	Active Encapsulation	Indication	Reference
Chitosan and PVA	Centrifugation molding using PDMS female molds	Anti-PD-L1 antibody and 1-methyl-D,L-tryptophan	Psoriasis	[111]
Carboxymethyl chitosan and *Bletilla striata* polysaccharide	Micromolding method	Triamcinolone acetonide and verapamil	Hypertrophic scar	[112]
PVP, PVA, sucrose	PLGA NP: emulsification and volatilization Keratin NP: chemical crosslinking MN: two-step template	Rapamycin-loaded PLGA NP and epigallocatechin gallate-keratin NP	Hair regrowth	[113]
Hyaluronic acid and hydroxypropyl-β-cyclodextrin	PLGA NP: nanoprecipitation MN: step-by-step molding centrifugation	Paclitaxel and indocyanine green porous PLGA NP	Cancer	[114]
CMC and trehalose	EV: mild sonication MNs: micromilling/spin-casting method	Curcumin and albumin extracellular vesicles	Inflammatory	[115,116]

PVA: polyvinyl alcohol; PDMS: polydimethylsiloxane; PVP: polyvinylpyrrolidone; PLGA NP: polylactic–glycolic acid copolymer nanoparticles; NP: nanoparticles; MN: microneedle; CMC: carboxymethyl cellulose; EV: extracellular vesicles.

## Data Availability

Not applicable.
